# Post-heating Fluorescence-based Alteration and Adulteration Detection of Extra Virgin Olive Oil

**DOI:** 10.1007/s10895-023-03165-8

**Published:** 2023-02-18

**Authors:** Omnia Hamdy, Haitham S. Mohammed

**Affiliations:** 1 Engineering Applications of Lasers Department, National Institute of Laser Enhanced Sciences, Cairo University, Giza, 12613 Egypt; 2 Biophysics Department, Faculty of Science, Cairo University, Giza, 12613 Egypt

**Keywords:** Food adulteration, Laser-induced fluorescence, Olive-oil, Heating

## Abstract

Olive oils are more expensive compared with other vegetable oils. Therefore, adulterating such expensive oil is prevalent. The traditional methods for olive oil adulteration detection are complex and require pre-analysis sample preparation. Therefore, simple and precise alternative techniques are required. In the present study, the Laser-induced fluorescence (LIF) technique was implemented for detecting alteration and adulteration of olive oil mixed with sunflower or corn oil based on the post-heating emission characteristics. Diode-pumped solid-state laser (DPSS, λ = 405 nm) was employed for excitation and the fluorescence emission was detected via an optical fiber connected to a compact spectrometer. The obtained results revealed alterations in the recorded chlorophyll peak intensity due to olive oil heating and adulteration. The correlation of the experimental measurements was evaluated via partial least-squares regression (PLSR) with an R-squared value of 0.95. Moreover, the system performance was evaluated using receiver operating characteristics (ROC) with a maximum sensitivity of 93%.

## Introduction

Olive oil is one of the vegetable oils which are very valued for the health of different organs especially the brain, the heart and the nervous system. It is rich in monounsaturated fatty acids, bioactive compositions, antioxidants, vitamins (K and E), and minerals [1]. Additionally, it needs minimum processing procedures for removing impurities and retaining its natural flavor. It is mechanically obtained from olive tree fruits and exposed to some treatment processes including washing, decantation, centrifugation, and filtration [2]. Commercially, the price of different types of olive oils is relatively higher than other vegetable oils such as soybean or sunflower oils. Therefore, adulteration of olive oil with these lower-priced oils is common and precise and fast techniques for detecting adulteration are encouraged. Currently, some chemical analyses based on gas-liquid chromatography are applied for the identification and quantification of adulterants in olive oils [3], however, most of the common chemical techniques are time consuming, costly, and require complex sample preparation in addition to the use of toxic chemicals [4, 5]. Alternatively, some analytical techniques including vibrational, chromatographic and thermal techniques can be utilized [6]. Moreover, spectroscopic techniques such as laser-induced breakdown spectroscopy [7, 8], Raman spectroscopy [9] and near-infrared spectroscopy [10] have been extensively utilized in the inspection of the quality and possible adulteration in different food products including olive oil [11–13].

Laser-induced fluorescence (LIF) spectroscopy is a very promising non-invasive optical modality utilized in various medical and industrial applications [14]. It has been applied for biological investigations [15, 16], tumor classification [17] and food adulteration detection [18] and quality inspection [8]. Regarding olive oil adulteration detection, LIF combined with different chemometric techniques has been widely employed to examine extra virgin olive oil blended with different vegetable oils [19]. It has been also utilized to monitor virgin olive oils at different storage conditions [20] and assess their quality [21, 22]. Nikolova et al. [23] analyzed the fluorescence spectra of olive oil adulterated with sunflower oil at different excitation wavelengths based on the tocopherol and fatty acid content using fiber optics spectrometer. Additionally, Mu et al. [24] proposed a portable system to detect and quantify extra virgin olive oil (EVOO) adulterated with rapeseed, peanut, and blend oils using 473-nm LIF. Recently, Zhang et al. [25] employed ultra-violet to blue LED-induced fluorescence spectroscopy to quantitatively detect adulteration in extra-virgin olive oil mixed with peanut oil, and soybean oil. Moreover, Abedin [26] studied the LIF spectra of various edible oils such as mustard, sunflower, corn, sesame, peanut, rice bran, flaxseed and olive to investigate their molecular features. LIF spectroscopic technique is preferred because it is safe, portable, needs almost no sample preparation and has relatively fast spectral acquisition. However, the conventional LIF scheme may encounter some noise that can overshadow its sensitivity and specificity [27].

The recently published results reveal that the storage period and freshness of olive oils affects its quality and hence affects the intensity of the fluorescence emission [28, 29]. For cooking purposes, olive oil could be heated above its smoke temperature [30]. Upon heating, some chemical reactions occurs including oxidation and polymerization which affect the olive oil nutritional compounds [31]. The thermal oxidation characteristics were utilized in quantifying the low concentration of adulterants (2% soybean oil) in extra-virgin olive based on LIF spectroscopy combined with chemometric analysis showing enhanced LIF spectra of the heated oil samples [32]. The present study has double aims; the first aim is to investigate the effect of heating on the florescence characteristics of EVOO. While the second objective is to employ the post-heating fluorescence modifications to detect adulteration in EVOO with sunflower and corn oils. To this end, the oil samples (pure and adulterated) were heated to smoke temperature (190 °C) and the resultant LIF spectra have been recorded and analyzed. The implemented method has been evaluated using receiver operating characteristic curve (ROC) and the experimental measurements have been statistically verified using partial least squares regression (PLSR) method.

## Methods 

### Laser-induced Fluorescence (LIF)

**Fig. 1:**
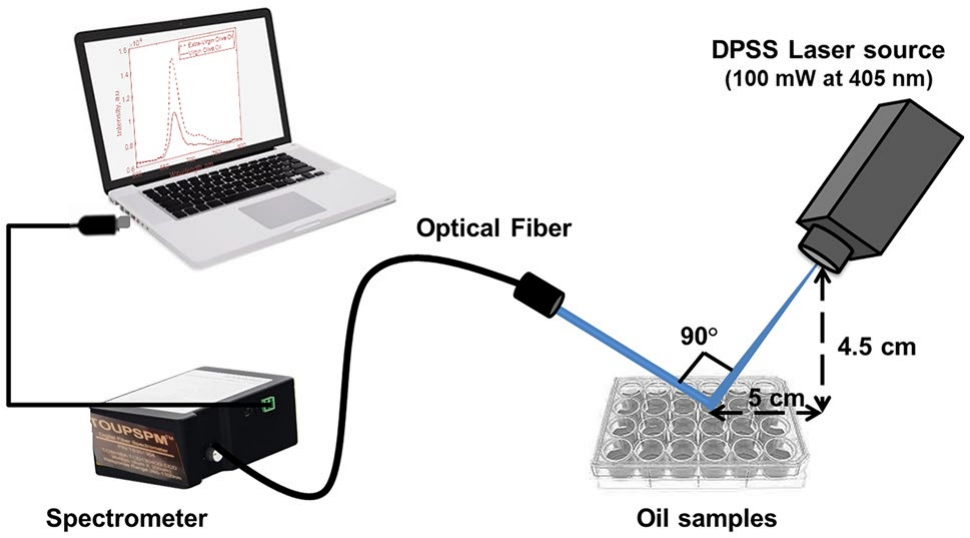
Schematic diagram of the optical setup used for excitation and recording of the oil samples' fluorescence spectra

The fluorescence measurements of different oil samples have been performed based on the optical configuration presented in Fig. 1. Due to the various fluorescence properties of olive oil components, different wavelengths could be used for excitation (typically from 270 to 458 nm) [33]. However, the 405-nm irradiation is commonly employed for obtaining the fluorescence emission of chlorophylls [26, 34, 35]. Therefore, a continuous-wave diode pumped solid-state (DPSS) laser source (XL-R405SD, Xinland international Co., Ltd, China) at wavelength of 405 nm and output power of 100 mW, has been used as the excitation source in the present work. The laser beam diameter is about 2 mm.

Upon interacting with the tested oil samples, the resultant fluorescence emission has been delivered to a digital spectrometer (STDFSM, Touptek Photonics Co. Ltd, Zhejiang China) via an optical fiber (200 µm core diameter and SMA 905 interface type). The distance between the laser source and the sample was identical to the distance between the sample and collecting fiber (~ 6.7 cm). The angle between the laser beam and the optical fiber with respect to the sample was 90°. The spectrometer was connected to the laptop computer through a USB cable. The measuring detector (Toshiba TCD1304AP Linear CCD array (Sony ILX511 2048 Linear CCD array)) has a 200-1100 nm response range, 3648 pixels and 8×200 µm pixel size. Data processing and analysis have been performed using the spectrometer software (Toup Spm) and Matlab R2018b.

### Sample Selection and Preparation

**Fig. 2:**
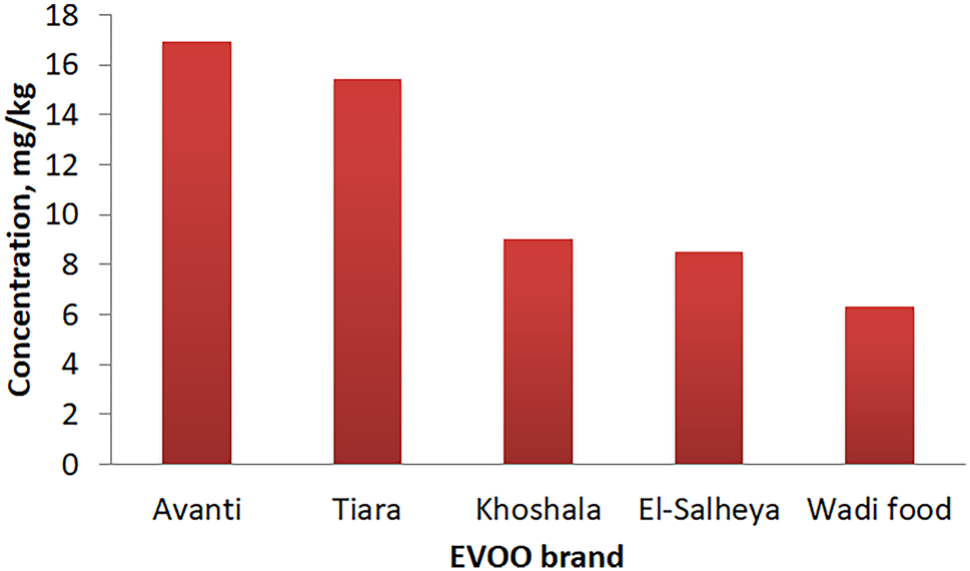
Chlorophyll-a contents of some common EVOO brands

Virgin olive oil (VOO), EVOO, sunflower oil, and corn oil samples have been purchased from different local hypermarkets. VOO and EVOO were initially investigated and the preliminary results revealed the higher fluorescence emission peak of the EVOO compared to VOO. Therefore, the rest of the experiments were performed using EVOO. To confirm that the EVOO samples purchased were definitely of extra virgin grade, the amount of chlorophyll-a in the tested EVOO (which was = 16.91 mg/kg) was measured using UV-VIS spectroscopy (UV-1750 spectrophotometer, Shimadzu, Kyoto, Japan). When compared to other commercially available brands in Egypt (such as Tiara, Khoshala, El-Salheya, and Wadi food, Avanti-branded EVOO had showed higher chlorophyll-a content as shown in Fig. 2 Therefore, it was chosen for the analysis.

A total 90 samples have been examined which were divided into 30 EVOO samples (brand name: Avanti) and 30 EVOO mixed with sunflower oil (brand name: Hla) and 30 EVOO mixed with corn oil (brand name: Roots), 1 mL each volume. The 30 samples of each oil category were identical products from the same brand that were purchased from several market places and had manufacturing dates that varied from January 2021 to March 2022. All the studied oil types were made in Egypt. The investigated samples were placed in a 24-wells plate using pipette. Sample heating has been achieved via a digital hotplate and a magnetic stirrer (MSH-10, DAIHAN Scientific - Korea). All samples were stored in the dark at room temperature 23 °C during the measurements time. The temperature during experiments was measured and controlled using a digital temperature controller and a thermocouple (XH-W3001, Generic, China).

### Partial Least Squares Regression (PLSR)

In the current investigations, LIF spectra of the examined oil samples were validated using the multivariate calibration technique Partial Least Squares Regression (PLSR). PLSR is applied to model response variable of highly correlated large number of predictor variables datasets via creating new predictors called “components” [36]. Unlike principle component regression (PCR) method, PLSR components that demonstrate the observed variability in the predictors have been constructed taking the response variable into account [37, 38]. Generally, the regression is used to determine the best-fit line to the dataset. Hence ensuring that the measured data is correlated and there are no odd values [39]. Based on a Matlab function named “plsregress”, PLSR of the obtained LIF spectra has been implemented in the current study. After defining the number of the PLS components, a partial least-squares regression of the response matrix on the predictor variables matrixes computed, then the predictor and response loadings were returned. Furthermore, R-squared value and root mean square error were estimated. In matrix representation, PLSR method is based on the following relations [40, 41]:

1 $$N={W}^{^{\prime}}\times Y$$

2 $$Y=P\times N+E$$

Substitute (2) in (1) gives:

3 $$Y=P\times {W}^{^{\prime}}\times Y+E=P\times W\times {}^{^{\prime}}Y+(I=P\times {W}^{^{\prime}})\times Y$$

where, N is the principal component, Y is the set of the observed responses, W is the set of the composing weights, P is the set of the principal component loadings and E is the residual variance. The singular value decomposition is then used for matrix solution without any matrix inversions as follows:

4 $$R={W}^{^{\prime}}\times D\times P$$

where, W is the left singular vectors orthonormal matrix with W×W’ = I (the identity matrix), P is the right singular values orthonormal matrix and D is the singular values diagonal matrix.

### Receiver Operator Curve (ROC)

ROC curve is obtained by plotting sensitivity versus “1- specificity” for classification purposes [42, 43]. It evaluates the performance of the applied method according to the acquired area under the ROC curve (AUC) [44, 45]. This curve is created based on “true positive, true negative, false positive, and false negative" concept [46]. In the present work, the ROC curves of the obtained results have been created using MATLAB in-house function. The LIF spectra of olive oil and olive oil mixed with sunflower or corn oils were used as the two classes of data for discrimination. At any point p, the test’s sensitivity (the true positive rate $TPR=\frac{true\_positive}{true\_positive+false\_negative}$) is represented by Se(p) = 1 − G(p) and its specificity (the false positive rate $FPR=\frac{false\_positive}{false\_positive+true\_negative}$) is given by Sp(p) = F(p). where, F and G are the distribution functions of X and Y which are two independent random variables denoting the measured data sets, respectively [47]. Then, the ROC curve is constructed by plotting the Se(p) versus 1−Sp(p) for -∞ ≤ p ≤ ∞.

## Results and Discussion

### LIF Spectra of Different Oil Samples

**Fig. 3:**
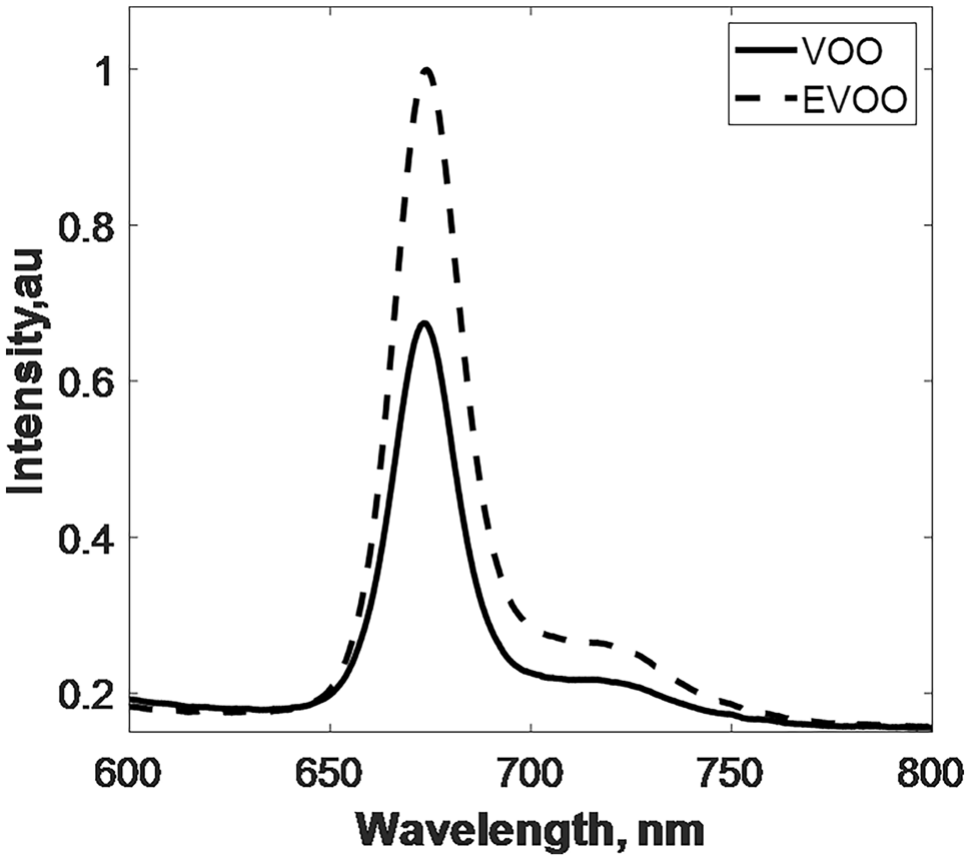
Laser-induced fluorescence spectra for virgin (VOO) and extra-virgin (EVOO) olive oils

LIF spectra of olive oils (VOO and EVOO) were investigated. The obtained Florescence spectra of 30 samples of EVOO and VOO samples were averaged and presented in Fig. 3.

**Fig. 4:**
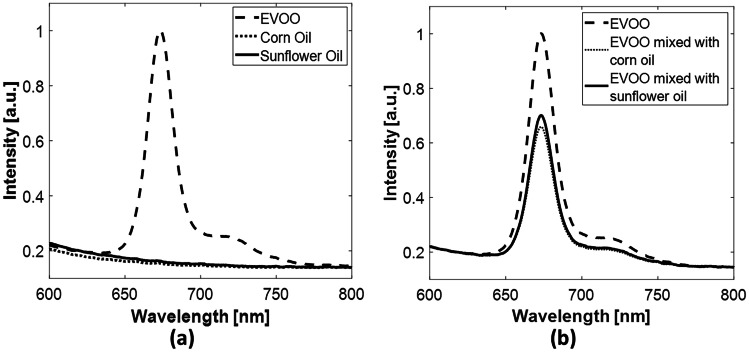
Laser-induced fluorescence spectra of (**a**) extra-virgin olive (EVOO), corn, and sunflower oils, (**b**) EVOO and EVOO oil mixed with corn or sunflower oil

The 675-nm emission peak of VOO is almost 33% lower than EVOO. Although, VOO and EVOO are made from olives, they are extracted via different methods. Therefore, they have different colors, tastes and nutritional benefits [48]. Moreover, relevant literature revealed that EVOO contains higher polyphenols and chlorophylls (and their derivatives) contents than VOO [49, 50] which may explain the lower 675-nm emission peak in VOO. Accordingly, experiments were performed using EVOO. Upon excitation with the incident laser light (405 nm), EVOO showed an emission peak at 675-nm which was associated with the presence of chlorophyll [51]. The fluorescence emission of EVOO in comparison with that of the corn and sunflower oils is illustrated in Fig. 4a. Additionally, variations in the recorded chlorophyll peak of unadulterated and adulterated EVOO are shown in Fig. 4b.

As demonstrated in Fig. 3a, the sunflower and corn oils have no fluorescence peaks. While in adulterated EVOO about 30% and 34% decrease in the overall fluorescence spectra has been observed for olive oil mixed with sunflower oil or corn oil respectively as presented in Fig. 4b.

Although it is documented in the literature that fluorescence emission of olive oil can be possible below 600 nm [23, 26], in the present investigations we only considered the change in the chlorophyll peak which is considered the highest recorded fluorescence peak (λ=675-nm) in olive oils [52]. Therefore, we specified the bandwidth to be from 600 – 800 nm.

### Post-heating LIF Spectra in Unadulterated and Adulterated Oil Samples

**Fig. 5:**
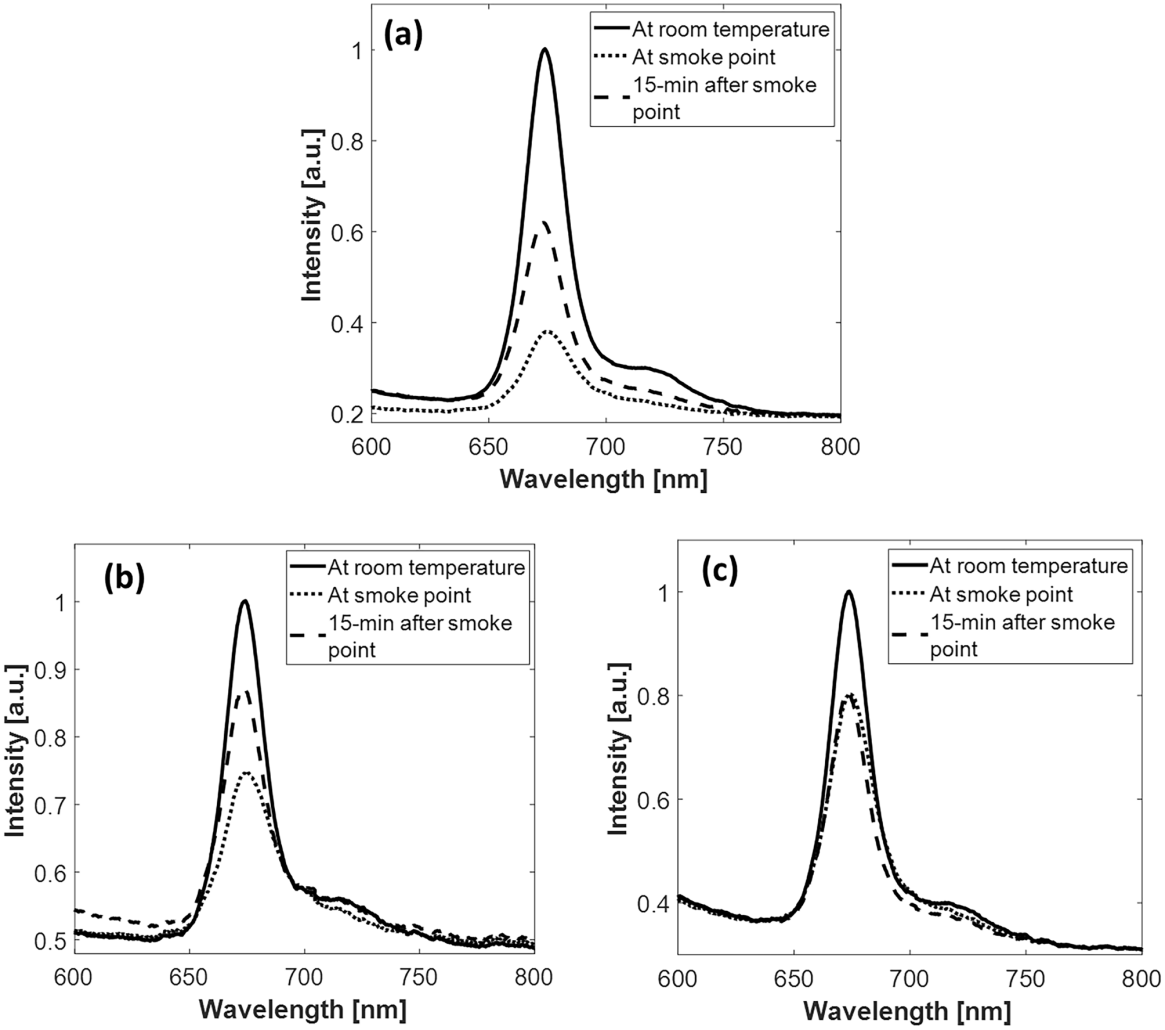
Fluorescence spectra of (**a**) EVOO (**b**) EVOO adulterated with corn oil (**c**) EVOO adulterated with sunflower oil before and after heating to the smoke point

EVOO samples were heated to its smoke point (about 190 °C) and their fluorescence spectra have been recorded and analyzed. As illustrated in Fig. 5a, the peak representing the chlorophyll (λ=675-nm) significantly decreased (about 62%) directly after heating., After 15 minutes the samples were returned to its initial temperate (23 °C) and the chlorophyll peak started to increase (61% recovery from original) however, it didn’t return to its normal value. On the other hand, the chlorophyll peak in EVOO adulterated with corn or sunflower oil attenuated by 30% and 20% respectively after heating to the smoke point as demonstrated in Fig. 5b and c, respectively. In the EVOO adulterated with corn oil, an increase (about 16%) in the fluorescence peak was noticed after the returning to its initial temperature (before heating). However, the fluorescence peak of the EVOO adulterated with sunflower oil remained constant and did not increase as occurred in the unadulterated EVOO or EVOO adulterated with corn oil. All the graphs were obtained utilizing 30 different samples (five replicates each) of the same blend (1:1 blending ratio) using different purchased products of the same brand.

**Fig. 6:**
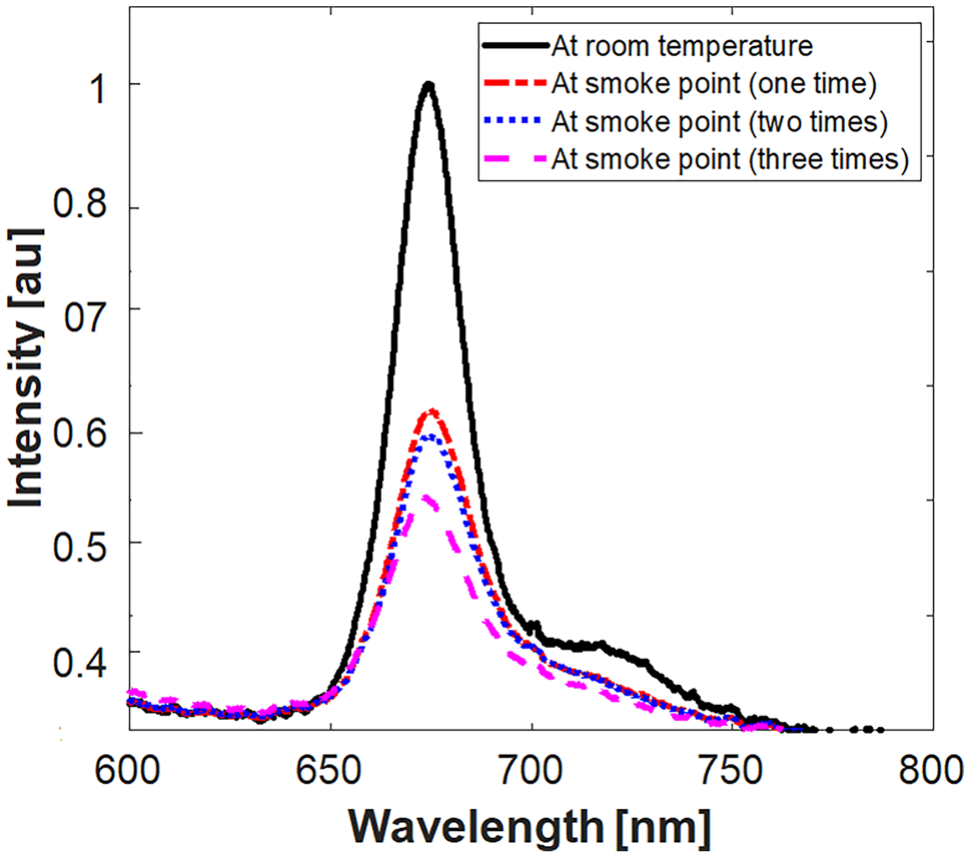
Effect of repeated heating on the chlorophyll peak in pure EVOO

Some physical changes such as viscosity increase or changing in color (darkening) may occur in vegetable oils after heating to a high temperature (i.e. >180 °C). Moreover, significant physical and chemical reactions including hydrolysis, oxidation, cyclization, and polymerization can be also arisen [53]. Such changes influence the sensory, taste, flavor and quality of the oils. Consequently, oil degradation resulting from heating to smoke point (thermal oxidation) may be responsible for the intensity reduction occurred in the recorded fluorescence peaks after heating [54]. Additionally, the repeated heating of EVOO (to the smoke point) revealed a continuous reduction in the chlorophyll peak in the recorded fluorescence emission as illustrated in Fig. 6. During this investigation, the temperature of the sample was recorded before the start of the experiment, and measurements were made after the heating process was complete before the samples were allowed to cool back to their initial temperature. Time interval for the whole process did not exceed 10 minutes.

In comparison with literature, Rasul and Inanc [55] showed that heating VOO to high temperatures (≥150 °C) results in a significant decrease in its chlorophyll content (about 99.5% after heating for 24 hr. at 200 °C). Moreover, Balaky et al. [56] studied the change in chlorophyll contents in addition to the oxidative stability in olive pomace oil due to different-period repetitive-heating procedures. Their results revealed a significant decrease (below 2.7%) in the chlorophyll contents after heating. However, in most of the previous studies, the change in the chlorophyll content was inspected based on analyzing the absorbance of the oil samples at different wavelengths using a spectrophotometer. Nevertheless, Cheikhousman et al. [52] have utilized fluorescence spectroscopy (excitation wavelengths at 330 and 450 nm) to monitor the deterioration occurred in EVOO upon heating [54]. Additionally, Saleem et al. [57] utilized the fluorescence spectroscopy (350-nm excitation source) to study the heating effect (from 140 to 180 °C) on EVOO to inspect its safety for cooking. Their results disclosed a decrease in the overall LIF intensity (including chlorophyll peak) after heating the oil samples above 150 °C, which agrees with our obtained results.

Although the present study aimed to investigate the post-heating chlorophyll fluorescence characteristics of unadulterated and adulterated EVOO, the use of a single excitation wavelength is considered a limitation of the present study. Consequently, further investigations for the post-heating fluorescence emission of EVOO are needed using other excitation wavelengths to analyze other contents rather than chlorophylls.

### PLSR of LIF Spectra

**Fig. 7:**
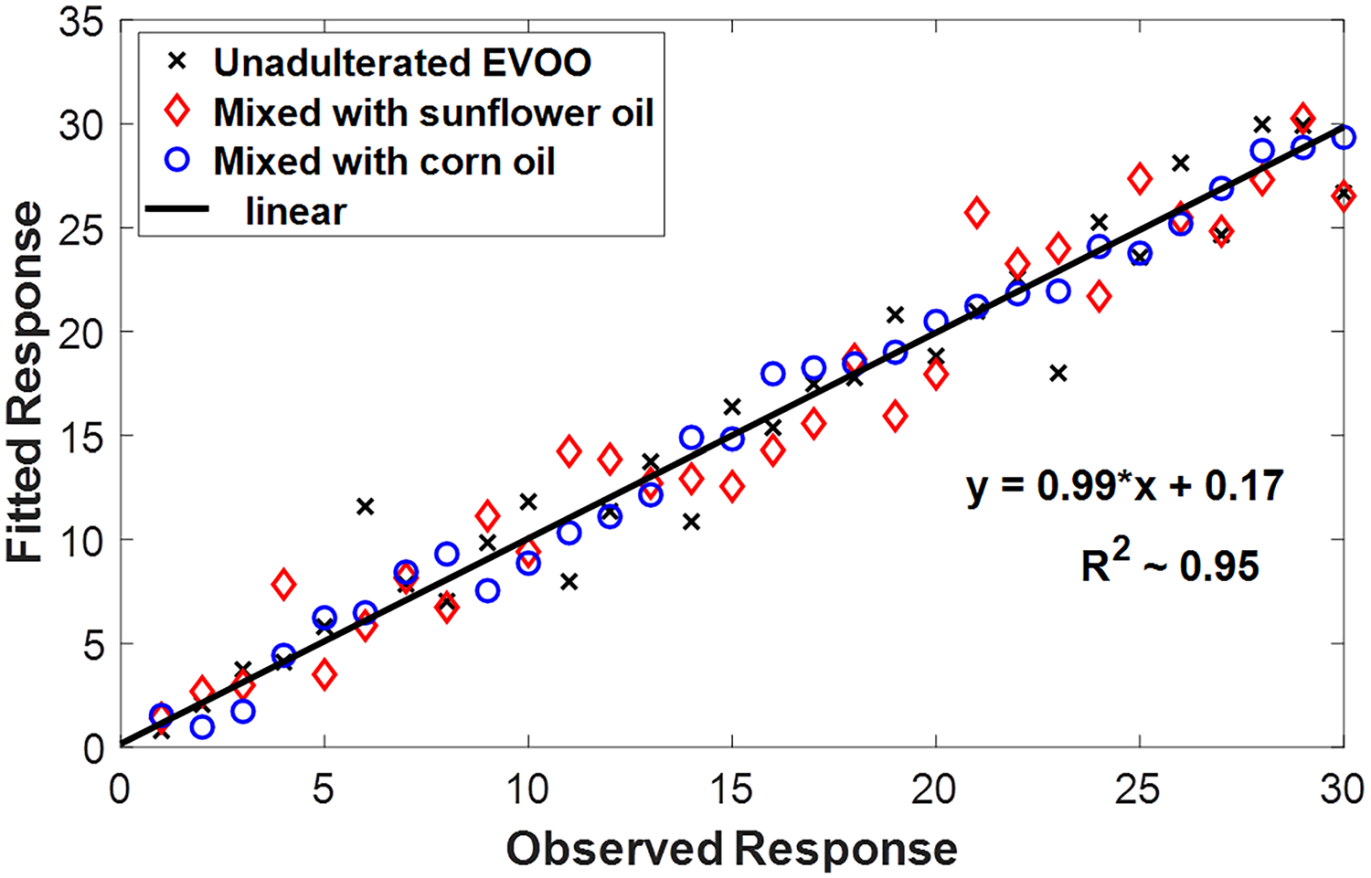
Partial Least Squares Regression (PLS) of the obtained LIF spectra of EVOO and EVOO adulterated with sunflower or corn oils

The correlation between the obtained LIF spectral measurements were statistically evaluated using the Partial Least Squares regression (PLSR) method. The loaded data in the PLSR model were the spectral intensities of the 30 samples unadulterated and adulterated (with corn or sunflower) EVOO, respectively. The “plsregress” Matlab function was used to fit the PLSR model with three PLS components. It is worthy to mention here that the number of components was specified according to the cross-validation method [58]. The predictive ability of the PLS model was assessed based on the coefficient of determination (R^2^) between observed data and fitted response values and the root mean square error (RMSE). Figure 7 shows the relation between the observed and predicted values based on our measured spectroscopic data. The calculated R^2^-value was 0.946 and the RMSE ~ 2.056 which indicate a good model performance for adulteration detection [59].

It is worthy to mention here that, PLSR was not implemented to reflect the detection performance of the proposed method. However, it employed to find the correlation between the obtained repeated spectral measurements. Other technique such as PCA has been widely utilized to statically analyze the adulteration detection models, which is not the aim of our study.

### ROC Curve Analysis

**Fig. 8:**
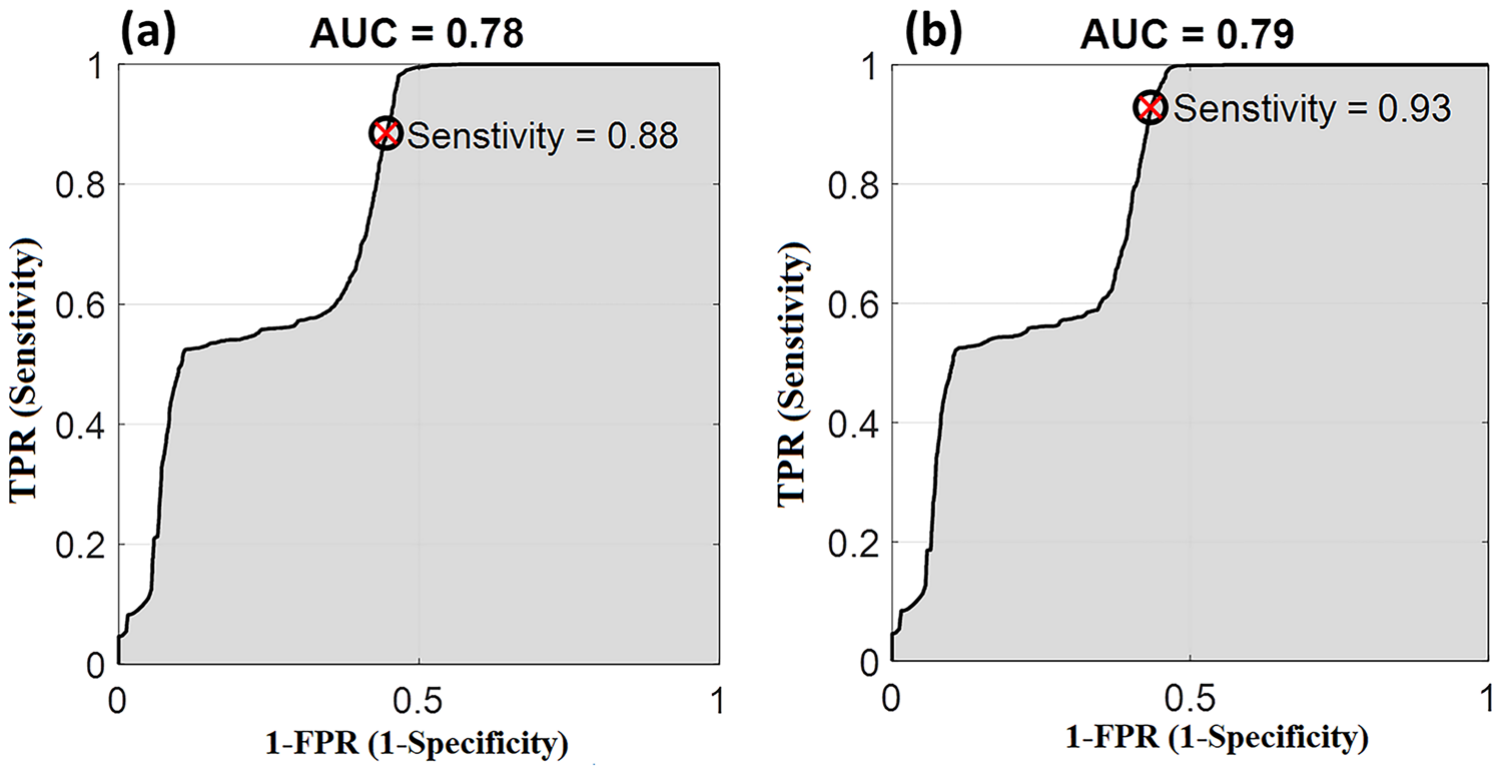
ROC curves for the discrimination between pure EVOO compared to (**a**) corn oil-adulterated EVOO (**b**) sunflower oil-adulterated EVOO at room temperature

The ROC curves for the discrimination between unadulterated and adulterated olive oil (with sunflower or corn oil) are presented in Fig. 8. The obtained ROC curves showed significant characteristics with sensitivity of 93%, 88%, for sunflower-adulterated EVOO and Corn-adulterated EVOO, respectively. The area under the curve (AUC) exceeds 75% in both cases. Such values are considered adequate for discrimination as revealed in the relevant literature [15, 44].

## Conclusions

In conclusion, olive oil alteration due to heating has been detected via LIF at 405 nm. The post-heating fluorescence spectra of adulterated and unadulterated oil samples are suggested to discriminate between different oil samples. After heating to the smoke point, a decrease in the chlorophyll fluorescence peak's intensity was detected in both the unadulterated EVOO (by 62%) and EVOO adulterated with equal ratio of corn or sunflower oil (by 30% and 20% respectively). Partial recovery of the fluorescence peak's intensity has been obtained by unadulterated and corn-adulterated EVOO. The correlation between the obtained repeated measurements were statistically assessed using PLSR method (R^2^ ~ 0.95 and RMSE ~ 2.056). Additionally, the sensitivity of the proposed method was evaluated using ROC curves giving 88% and 0.93% for EVOO adulterated with corn oil and EVOO adulterated with sunflower oil respectively. The present work provided a simple, portable, sensitive, and rapid method for detecting alteration and adulteration of EVOO.

## Data Availability

This manuscript has no associated data.
